# ﻿Three new species of the spider genus *Utivarachna* Kishida, 1940 (Araneae, Trachelidae) from China and Vietnam

**DOI:** 10.3897/zookeys.1181.110628

**Published:** 2023-10-06

**Authors:** Chang Chu, Shuqiang Li, Dinh-Sac Pham, Zhiyuan Yao

**Affiliations:** 1 College of Life Science, Shenyang Normal University, Shenyang 110034, Liaoning, China Shenyang Normal University Shenyang China; 2 Institute of Zoology, Chinese Academy of Sciences, Beijing 100101, China Institute of Zoology, Chinese Academy of Sciences Beijing China; 3 Vietnam National Museum of Nature (VNMN), Vietnam Academy of Science and Technology (VAST), 18 Hoang Quoc Viet, Cau Giay, Hanoi, Vietnam Vietnam National Museum of Nature Hanoi Vietnam

**Keywords:** biodiversity, morphology, Southeast Asia, taxonomy, trachelid

## Abstract

Three new species belonging to the *kinabaluensis* group of the trachelid genus *Utivarachna* Kishida, 1940 are reported from China and Vietnam: *U.linyejiei***sp. nov.** (♂♀), *U.tamdao***sp. nov.** (♂♀), and *U.zhengguoi***sp. nov.** (♂♀). Type specimens are deposited in the Institute of Zoology, Chinese Academy of Sciences (IZCAS) in Beijing, China and the Vietnam National Museum of Nature (VNMN) in Hanoi, Vietnam.

## ﻿Introduction

The family Trachelidae Simon, 1897 is a small spider group which currently contains 20 genera and 266 species (Marusik and Fomichev 2020; WSC 2023). It is distributed worldwide but occurs predominately in Africa and the Americas (Rivera-Quiroz and Álvarez-Padilla 2015; Quasin et al. 2018; Ono and Aung 2022; WSC 2023). Of these, only 34 and one known species recorded from China and Vietnam, respectively (Li et al. 2022; Lin et al. 2023; WSC 2023). Trachelid spiders occupy a wide range of habitats in a variety of ecosystems, including under loose bark of trees, in rolled leaves, under stones, in leaf litter, in wood debris, on the forest floor, in marshes, and in houses (Haddad and Lyle 2008; Quasin et al. 2018; González Márquez et al. 2021; Zhang et al. 2022).

*Utivarachna* Kishida, 1940 is a relatively small trachelid genus, with 24 described species distributed in South and Southeast Asia (Li et al. 2022; WSC 2023). Among them, nine and one species have been recorded from China and Vietnam, respectively (Zhu et al. 1998; Zhao and Peng 2014; Jin et al. 2015; Liu et al. 2020; Li et al. 2022; Lin et al. 2023; WSC 2023). This genus is composed of four species groups: the *dusun* group, the *fukasawana* group, the *kinabaluensis* group, and the *phyllicola* group (Deeleman-Reinhold 2001; Liu et al. 2020). The *kinabaluensis* group includes 15 known species, making it the most species-rich group in the genus (Dankittipakul et al. 2011; Liu et al. 2020; Li et al. 2022; Lin et al. 2023). Members of this species group can often be found in forest leaf litter (Deeleman-Reinhold 2001; Liu et al. 2020).

In the present study, we describe three new species based on males and females from China and Vietnam which are assigned to the *kinabaluensis* group.

## ﻿Materials and methods

Specimens were examined and measured with a Leica M205 C stereomicroscope. Left male palps and epigynes were photographed. Vulvae were treated in a warm 10% potassium hydroxide (KOH) solution to dissolve soft tissues before illustration. Images were captured with a Canon EOS 750D wide zoom digital camera (24.2 megapixels) mounted on the stereomicroscope mentioned above, and assembled using Helicon Focus v. 3.10.3 image-stacking software (Khmelik et al. 2005). All measurements are given in millimetres (mm). Leg measurements are shown as: total length (femur, patella, tibia, metatarsus, tarsus). Leg segments were measured on their dorsal side. The species distribution map was generated with ArcGIS v. 10.2 (ESRI Inc.). Type specimens are deposited in the Institute of Zoology, Chinese Academy of Sciences (**IZCAS**) in Beijing, China and the Vietnam National Museum of Nature (**VNMN**) in Hanoi, Vietnam.

Terminology and taxonomic descriptions follow Zhao and Peng (2014), Jin et al. (2015), and Liu et al. (2020).

The following abbreviations are used in the descriptions:

**AER** anterior eye row;

**ALE** anterior lateral eye;

**AME** anterior median eye;

**MOA** median ocular area;

**PER** posterior eye row;

**PLE** posterior lateral eye;

**PME** posterior median eye;

**RTA** retrolateral tibial apophysis.

## ﻿Taxonomy

**Family Trachelidae Simon, 1897**

### ﻿Genus Utivarachna

Taxon classificationAnimaliaAraneaeTrachelidae

Kishida, 1940

A045BB38-0230-5628-B723-73D939414F37

#### Type species.

*Utivarachnafukasawana* Kishida, 1940 from Borneo.

#### Composition.

*Utivarachna* includes 24 species distributed in South and Southeast Asia. Of these, 10 species are distributed in China and Vietnam: *U.arcuata* Zhao & Peng, 2014 (♂♀) from China, *U.fabaria* Zhao & Peng, 2014 (♂♀) from China, *U.fanjing* Li, Zhang & Yu, 2022 (♂♀) from China, *U.gongshanensis* Zhao & Peng, 2014 (♀) from China, *U.gui* (Zhu, Song & Kim, 1998) (♂♀) from China, *U.lata* Jin, Yin & Zhang, 2015 (♂♀) from China, *U.subfabaria* Liu, Xu & Haddad, 2020 (♂♀) from China, *U.taiwanica* (Hayashi & Yoshida, 1993) (♂) from China, *U.tangi* Liu, Xu & Haddad, 2020 (♀) from China, *U.yumaoi* Lin & Li, 2023 (♂) from Vietnam.

### ﻿ Utivarachna linyejiei

Taxon classificationAnimaliaAraneaeTrachelidae

Chu & Li sp. nov.

C5781BDF-EE52-57F4-BD50-B9AC62D56C9D

https://zoobank.org/3D93883A-AFDF-445F-AF51-670383CE9135

Figs 1 , 2 , 3

#### Type materials.

***Holotype*** ♂ (IZCAS-Ar44626): **Vietnam**: Vinh Phuc Province: Tam Dao National Park, disturbed forest (21.5209°N, 105.5583°E, 693 m a.s.l.), hand caught in leaf litter, 12.XII.2007, leg. Dinh-Sac Pham. ***Paratypes***: 1♂ (IZCAS-Ar44627), 1♂ (VNMN) and 1♀ (IZCAS-Ar44629), same data as holotype.

#### Etymology.

The specific name is dedicated to Mr Yejie Lin, who has helped us greatly with this research; noun (name) in genitive case.

#### Diagnosis.

**Figure 1.**
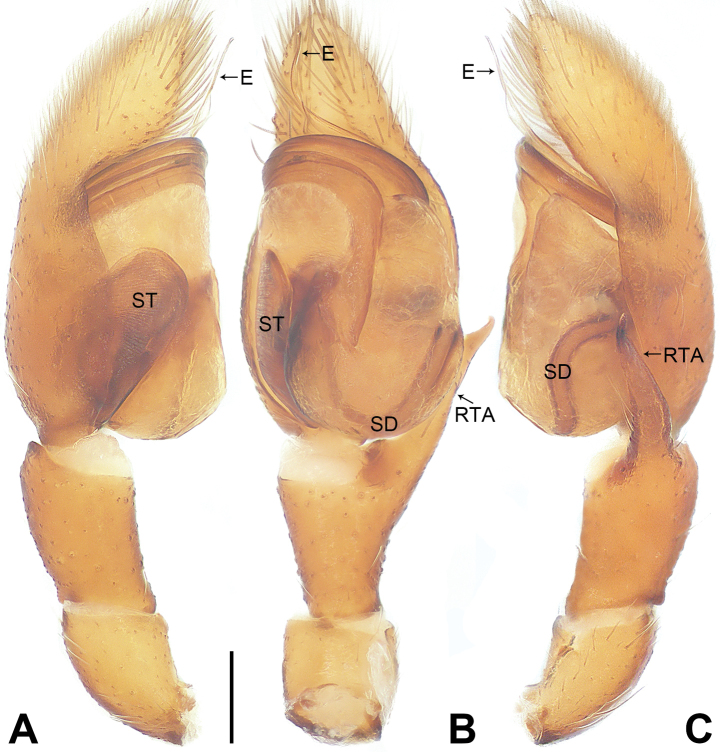
*Utivarachnalinyejiei* sp. nov., holotype male **A–C** palp **A** prolateral view **B** ventral view **C** retrolateral view. Abbreviations: E = embolus, RTA = retrolateral tibial apophysis, SD = sperm duct, ST = subtegulum. Scale bar: 0.20 mm.

**Figure 2.**
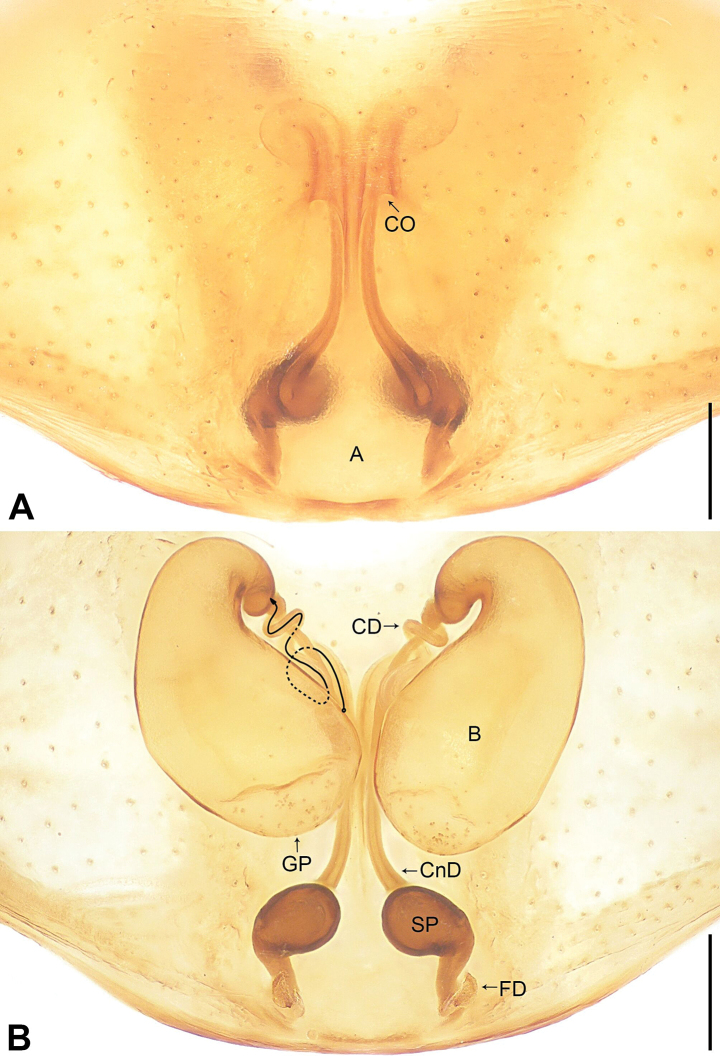
*Utivarachnalinyejiei* sp. nov., paratype female **A** epigyne, ventral view **B** vulva, dorsal view. Abbreviations: A = atrium, B = bursa, CD = copulatory duct, CnD = connecting duct, CO = copulatory opening, FD = fertilization duct, GP = glandular particles, SP = spermathecae. Scale bars: 0.20 mm.

**Figure 3.**
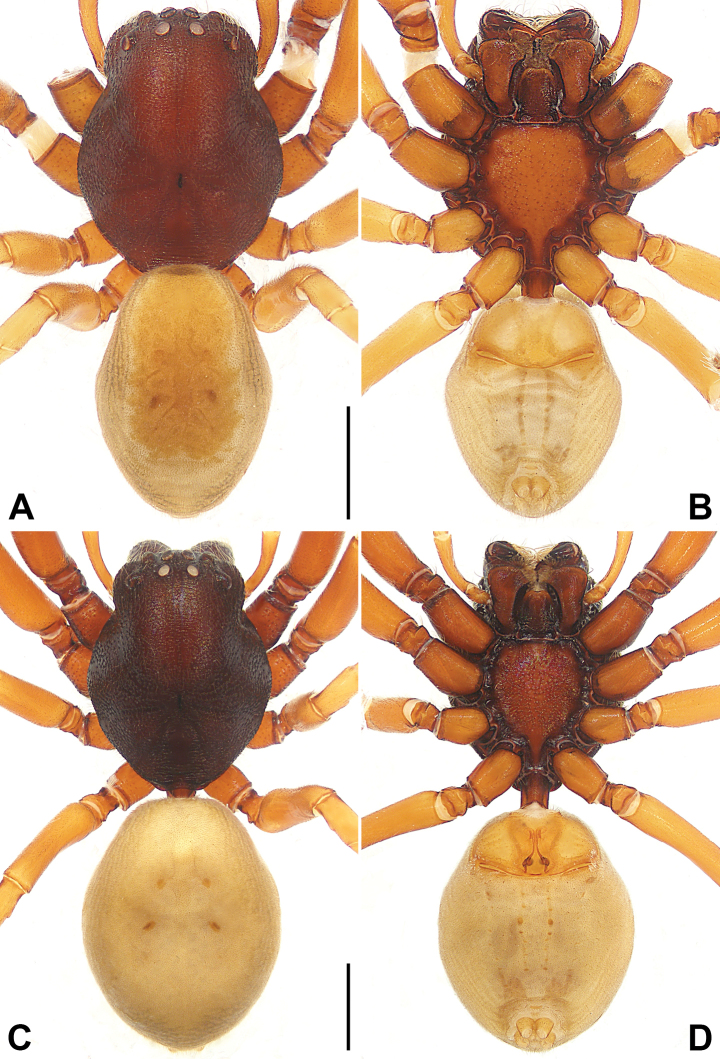
*Utivarachnalinyejiei* sp. nov., holotype male (**A, B**) and paratype female (**C, D**) **A–D** habitus **A** dorsal view **B** ventral view **C** dorsal view **D** ventral view. Scale bars: 1.00 mm.

The new species resembles *U.fabaria* Zhao & Peng, 2014 (cf. Figs 1–3 and Jin et al. 2015: 573, figs 4–6), as males have a similar long RTA (Fig. 1B, C), and females have a nearly trapezoidal atrium (A) (Fig. 2A), bean-shaped bursae (B) (Fig. 2B), and laminar fertilization ducts (FD) (Fig. 2B). Males can be distinguished by the terminal portion of embolus slightly twist, almost reaching cymbium distally (Fig. 1B; vs terminal portion of embolus straight, subdistally reaching cymbium in *U.fabaria*), by the short subtegulum (ST), which does not reach the embolus (E) in ventral view (Fig. 1B; vs subtegulum long, almost reaching embolus in ventral view in *U.fabaria*), and by the sperm duct (SD) extending to the base of tegulum (Fig. 1B; vs sperm duct separated from the base of tegulum by nearly three times the width of the sperm duct in *U.fabaria*). Females can by distinguished by the copulatory openings (CO) transverse, separated by about three times their diameter (Fig. 2A; vs copulatory openings oblique, separated by less than their diameter in *U.fabaria*), by the copulatory ducts (CD) strongly convoluted, basal and middle part with two twists, distal part coiled around connecting duct (CnD) (Fig. 2B; vs copulatory ducts not twisted in *U.fabaria*), by the connecting ducts located on the area between copulatory openings (Fig. 2A, B; vs connecting ducts located on the lateral areas of copulatory openings in *U.fabaria*), by the posterior part of bursae wider than middle part of it (Fig. 2B; vs posterior part of bursae as wide as middle part of it in *U.fabaria*), and by the spermathecae (SP) separated by about half of their diameter (Fig. 2B; vs spermathecae separated by less than half of their diameter in *U.fabaria*).

#### Description.

**Male.** Habitus (Fig. 3A, B). Total length 4.59. Carapace (Fig. 3A): length 2.32, width 1.82, reddish brown; cervical groove, radial grooves indistinct; fovea distinct, dark, and short. Eyes (Fig. 3A): AER procurved, PER recurved in dorsal view, PER wider than AER. Eye sizes and interdistances: AME 0.12, ALE 0.15, PME 0.13, PLE 0.15; AME–AME 0.07, AME–ALE 0.11, PME–PME 0.17, PME–PLE 0.24; MOA 0.35 long, anterior width 0.30, posterior width 0.44. Mouthparts (Fig. 3B): chelicerae reddish brown, with three promarginal (middle largest) and four retromarginal (proximal largest, distal smallest) teeth; endites depressed posteriorly, slightly convergent anteriorly, with dense setae on inner margin; labium nearly trapezoidal, length 0.49, width 0.38. Sternum (Fig. 3B) length 1.30, width 1.14, light reddish brown, with reddish-brown edges, with precoxal triangles and intercoxal extensions, posterior region protruding strongly between coxae IV. Pedicel cylindrical, sclerotized, relatively short, reddish brown. Abdomen (Fig. 3A, B) faintly yellow, 2.27 long, 1.56 wide, dorsum with scutum covering more than half of dorsal surface, with four indistinct reddish-brown spots centrally; venter with brown stripes, two lines of spots in the median field. Spinnerets surrounded by brown rings. Legs: anterior legs reddish brown, distinctly thicker than yellowish-brown posterior legs. Leg measurements: I 6.33 (1.89, 0.88, 1.55, 1.27, 0.74); II 5.81 (1.75, 0.74, 1.40, 1.26, 0.66); III 4.24 (1.22, 0.59, 0.83, 1.11, 0.49); IV 5.49 (1.56, 0.62, 1.19, 1.53, 0.59).

***Palp*** (Fig. 1A–C): tibia shorter than half of cymbium length; RTA about 1.08 times longer than tibia, with wide base and narrow, blunt tip, with slight curvature distally. Bulb nearly oval, posterior part wider than anterior part; tegulum approximately 1.46 times as long as its maximum width in ventral view; subtegulum (ST) sclerotized, occupying approximately 1/5 of tegulum width in ventral view; sperm duct (SD) distinct, U-shaped in ventral view, extending to base of tegulum. Embolus (E) long, anticlockwise, obliquely coiled twice, coils as wide as minimum width of tegulum; basal portion of embolus lamellar, wide, arising at 12:30 o’clock from bulb; terminal portion of embolus filiform, slightly twist, suspended in above distal cymbial alveolus.

**Female.** Habitus (Fig. 3C, D). As in male except as noted. Total length 5.85. Carapace length 2.84, width 2.16, dark reddish brown. Eye (Fig. 3C) sizes and interdistances: AME 0.14, ALE 0.15, PME 0.13, PLE 0.15; AME–AME 0.09, AME–ALE 0.13, PME–PME 0.20, PME–PLE 0.33; MOA 0.41 long, anterior width 0.35, posterior width 0.46. Mouthparts (Fig. 3D): chelicerae with three promarginal (middle largest) and four retromarginal (proximal largest, distal smallest) teeth. Sternum (Fig. 3D) length 1.55, width 1.34, reddish brown with dark reddish-brown edges. Abdomen (Fig. 3C, D): length 3.01, width 2.34, dorsum with four distinct, reddish-brown spots centrally. Leg measurements: I 7.36 (2.16, 0.94, 1.81, 1.58, 0.87); II 7.10 (2.12, 0.91, 1.65, 1.57, 0.85); III 5.36 (1.53, 0.76, 1.10, 1.34, 0.63); IV 7.02 (2.00, 0.76, 1.60, 1.99, 0.67).

***Epigyne*** (Fig. 2A, B): epigynal plate longer than wide, spermathecae (SP) distinct, and bursae (B) indistinct in ventral view. Atrium (A) large and nearly trapezoidal, occupying more than half of length of epigyne, posterior margin wider than anterior margin. Copulatory openings (CO) small, semicircular, located at submedially, separated by about three times their diameter. Copulatory ducts (CD) long, strongly convoluted, basal and middle part with two twists, distal part coiled around connecting duct (CnD). Connecting ducts thin and slender, located on the area between copulatory openings, separated by less than spermathecae diameter. Bursae nearly bean-shaped, anterior part strongly constricted and curved, posterior part five times width of anterior part; bursae with several small clusters of glandular particles (GP) on posterior surface, occupying about 1/8 of bursa diameter. Spermathecae elliptical, small, separated by less than their diameter. Fertilization ducts (FD) laminar, separated from each other by posterior width of atrium.

#### Distribution.

Vietnam (Vinh Phuc, type locality; Fig. 10).

### ﻿ Utivarachna tamdao

Taxon classificationAnimaliaAraneaeTrachelidae

Chu & Li sp. nov.

9E6F30D6-05C1-52EA-BF31-E24548C2B168

https://zoobank.org/7CC2C53C-5BBB-49BD-A81E-13835F82B7E5

Figs 4 , 5 , 6

#### Type materials.

***Holotype*** ♂ (IZCAS-Ar44630): **Vietnam**: Vinh Phuc Province: Tam Dao National Park, natural forest (21.4872°N, 105.6201°E, 870 m a.s.l.), hand caught in leaf litter, 18.IX.2007, leg. Dinh-Sac Pham. ***Paratypes***: 1♂ (IZCAS-Ar44631) and 2♀ (IZCAS-Ar44632, 44633), same data as holotype.

#### Etymology.

The specific name is named after type locality; noun in apposition.

#### Diagnosis.

**Figure 4.**
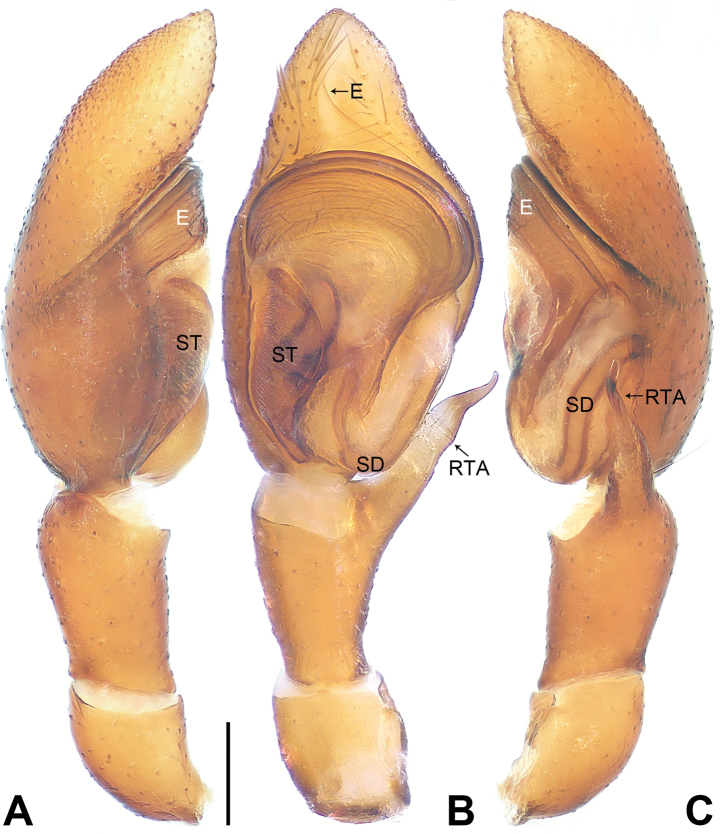
*Utivarachnatamdao* sp. nov., holotype male **A–C** palp **A** prolateral view **B** ventral view **C** retrolateral view. Abbreviations: E = embolus, RTA = retrolateral tibial apophysis, SD = sperm duct, ST = subtegulum. Scale bar: 0.20 mm.

**Figure 5.**
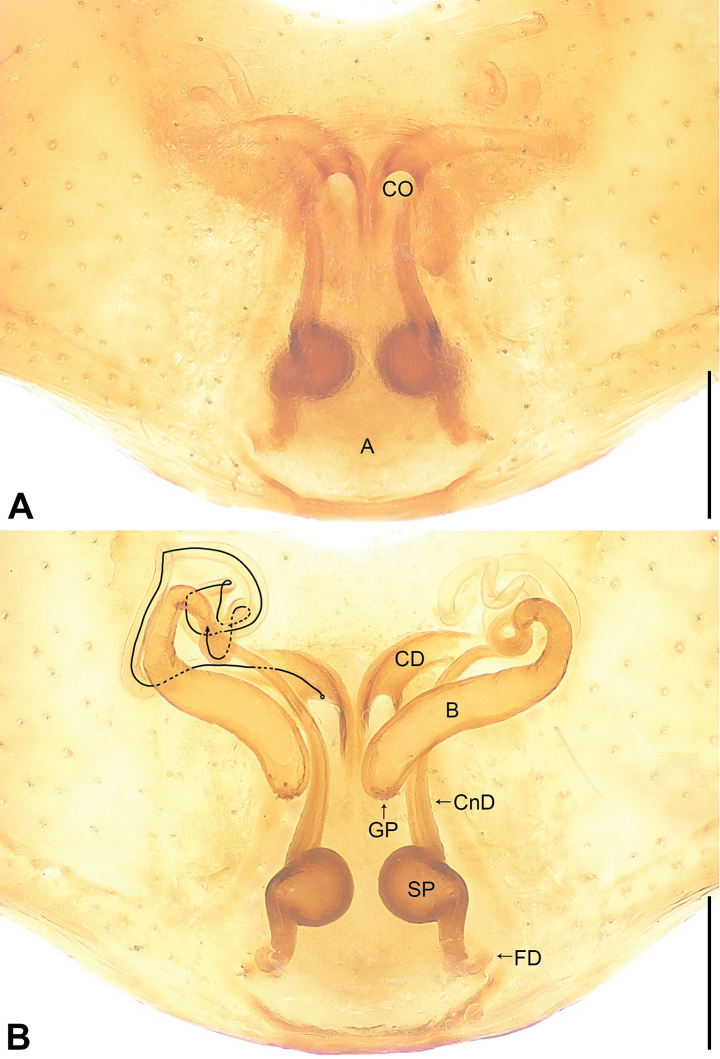
*Utivarachnatamdao* sp. nov., paratype female **A** epigyne, ventral view **B** vulva, dorsal view. Abbreviations: A = atrium, B = bursa, CD = copulatory duct, CnD = connecting duct, CO = copulatory opening, FD = fertilization duct, GP = glandular particles, SP = spermathecae. Scale bars: 0.20 mm.

**Figure 6.**
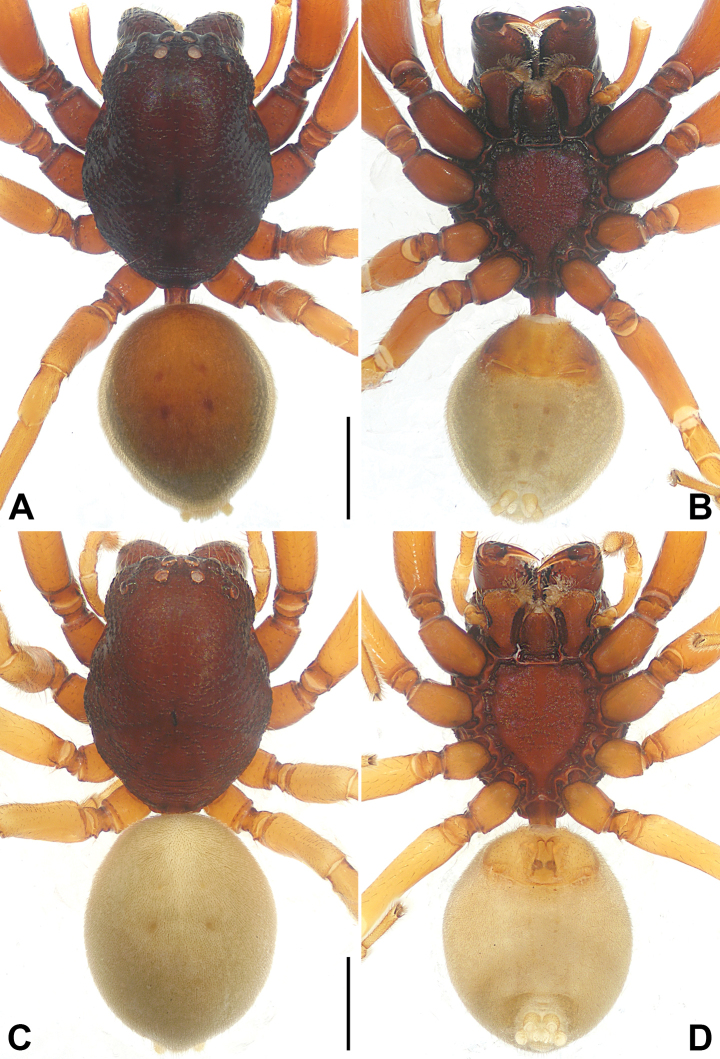
*Utivarachnatamdao* sp. nov., holotype male (**A, B**) and paratype female (**C, D**) **A–D** habitus **A** dorsal view **B** ventral view **C** dorsal view **D** ventral view. Scale bars: 1.00 mm.

The new species resembles *U.kinabaluensis* Deeleman-Reinhold, 2001 (cf. Figs 4–6 and Deeleman-Reinhold 2001: 381, figs 593–597), as males have a similar U-shaped sperm duct (SD) (Fig. 4B), the tegulum is widest in anterior part (Fig. 4B), the embolus (E) is coiled (Fig. 4A–C), females have a nearly trapezoidal atrium (A) (Fig. 5A), connecting ducts (CnD) are thin and slender (Fig. 5B), and fertilization ducts (FD) are laminar (Fig. 5B). Males can be distinguished in having the RTA widest at its base (Fig. 4B, C; vs RTA widest at middle, distally with hook-shaped apophysal claw in *U.kinabaluensis*). Females can be distinguished from *U.kinabaluensis* in having the copulatory openings (CO) located anteriorly (Fig. 5A; vs posteriorly), the atrium large, occupying about 2/3 of the epigyne length (vs atrium small, occupying about 1/4 of the epigyne length), the copulatory ducts (CD) forming irregular loops and these loops located anteriorly (Fig. 5B; vs copulatory ducts regularly coiled and these coils medially located), the bursae (B) located between the copulatory ducts and spermathecae (SP), anterior part strongly constricted and curved, and posterior part three times width of anterior part (Fig. 5B; vs bursae located in anterior of copulatory ducts, base to middle part strongly constricted and curved, posterior part five times the width of the anterior part), and the spermathecae globular (Fig. 5B; vs gourd-shaped). The new species also resembles *U.lata* Jin, Yin & Zhang, 2015 (cf. Figs 4–6 and Jin et al. 2015: 570, figs 1–3) as males have a similar U-shaped sperm duct (Fig. 4B), leaf-shaped subtegulum (ST) in ventral view (Fig. 4B), and long RTA (Fig. 4B, C), and females have similar copulatory openings located anteriorly (Fig. 5A), a large atrium (Fig. 5A), and globular spermathecae (Fig. 5B). Males can be distinguished by the oval bulb, which is widest in anteriorly (Fig. 4B; vs almost square in *U.lata*) and by the curved basal portion of the embolus (Fig. 4B, C; vs oblique in *U.lata*). Females can by distinguished by the copulatory openings width/atrium posterior width: 1/6 (Fig. 5A; vs copulatory openings width/atrium posterior width: 1/2 in *U.lata*), by the copulatory ducts forming irregular loops (Fig. 5B; vs copulatory ducts coiled three times around the anterior of connecting duct in *U.lata*), and by the bursae anterior part strongly constricted and curved (Fig. 5B; vs bursae anterior part slightly constricted and curved, posterior end very close to spermathecae in *U.lata*).

#### Description.

**Male.** Habitus (Fig. 6A, B). Total length 4.68. Carapace (Fig. 6A): length 2.48, width 1.86, deep reddish brown; cervical groove, radial grooves, and fovea indistinct. Eyes (Fig. 6A): AER procurved, PER recurved in dorsal view, PER wider than AER. Eye sizes and interdistances: AME 0.15, ALE 0.13, PME 0.13, PLE 0.14; AME–AME 0.06, AME–ALE 0.12, PME–PME 0.19, PME–PLE 0.27; MOA 0.36 long, anterior width 0.34, posterior width 0.46. Mouthparts (Fig. 6B): chelicerae deep reddish brown, with three promarginal (middle largest) and five retromarginal (proximal largest, distal smallest) teeth; endites depressed posteriorly, slightly convergent anteriorly, with dense setae on inner margin; labium nearly trapezoidal, length 0.47, width 0.39. Sternum (Fig. 6B) length 1.29, width 0.97, deep reddish-brown, with dark edges, with precoxal triangles and intercoxal extensions, posterior region protruding strongly between coxae IV. Pedicel cylindrical, sclerotized, relatively short, reddish brown. Abdomen (Fig. 6A, B) grey, 2.20 long, 1.75 wide, dorsum with scutum covering more than half of dorsal surface, with four brown central spots; venter with indistinct two lines of spots in the median field. Spinnerets yellow. Legs: anterior legs reddish brown, distinctly thicker than yellowish-brown posterior legs. Leg measurements: I 6.03 (1.83, 0.77, 1.55, 1.18, 0.70); II 4.82 (1.62, 0.71, 0.89, 1.07, 0.53); III 4.37 (1.26, 0.62, 0.89, 1.07, 0.53); IV 5.50 (1.52, 0.67, 1.20, 1.52, 0.59).

***Palp*** (Fig. 4A–C): tibia shorter than half of cymbium length; RTA about 0.98 times longer than tibia, distinctly narrow subdistally to distally, with distinct anterior curvature distally. Bulb oval, widest in anterior part; tegulum approximately 1.39 times as long as its maximum width in ventral view; subtegulum (ST) sclerotized, occupying approximately 1/3 of tegulum width in ventral view; sperm duct (SD) distinct, U-shaped in ventral view, extending to base of tegulum. Embolus (E) long, anticlockwise, obliquely coiled twice, coils as wide as maximum width of tegulum; basal portion of embolus lamellar, wide, arising at 2:30 o’clock from bulb; terminal portion of embolus filiform, resting in distal cymbial alveolus.

**Female.** Habitus (Fig. 6C, D). As in male except as noted. Total length 5.34. Carapace length 2.70, width 2.02, reddish brown; fovea distinct, dark, and short. Eye (Fig. 6C) sizes and interdistances: AME 0.12, ALE 0.13, PME 0.13, PLE 0.13; AME–AME 0.09, AME–ALE 0.12, PME–PME 0.21, PME–PLE 0.25; MOA 0.32 long, anterior width 0.31, posterior width 0.44. Mouthparts (Fig. 6D): chelicerae with three promarginal (middle largest) and five retromarginal (proximal largest, distal smallest) teeth. Sternum (Fig. 6D) length 1.50, width 1.17, reddish brown. Abdomen (Fig. 6C, D): length 2.54, width 2.08, dorsum with four central, indistinct, reddish-brown spots; venter without pattern. Spinnerets surrounded by brown rings. Leg measurements: I 5.56 (1.64, 0.75, 1.32, 1.12, 0.73); II 5.24(1.54, 0.69, 1.19, 1.14, 0.68); III 4.41 (1.24, 0.63, 0.90, 1.07, 0.57); IV 5.73 (1.57, 0.66, 1.27, 1.58, 0.65).

***Epigyne*** (Fig. 5A, B): epigynal plate longer than wide, spermathecae distinct and bursae indistinct in ventral view. Atrium (A) large and nearly trapezoidal, occupying about 2/3 of epigyne length, posterior margin wider than anterior margin. Copulatory openings (CO) semicircular, located at anteriorly, separated by about their diameter. Copulatory ducts (CD) long, anterior part wide and posterior part narrow; copulatory ducts convoluted posteriorly, forming irregular loops. Connecting ducts (CnD) thin and slender, located on lateral areas of copulatory openings, separated by more than spermathecae (SP) diameter. Bursae (B) nearly rod-shaped, anterior part strongly constricted and curved, posterior part three times width of anterior part; bursae with several small clusters of glandular particles (GP) on surface of distal margin. Spermathecae globular, separated by less than half of their diameter. Fertilization ducts (FD) laminar, separated from each other by posterior width of atrium.

#### Distribution.

Vietnam (Vinh Phuc, type locality; Fig. 10).

### ﻿ Utivarachna zhengguoi

Taxon classificationAnimaliaAraneaeTrachelidae

Chu & Li sp. nov.

17614417-E417-5071-997F-BA745B8C6D37

https://zoobank.org/10E3A567-A9C3-4A37-A1CC-13063399D495

Figs 7 , 8 , 9

#### Type materials.

***Holotype*** ♂ (IZCAS-Ar44634): **China**: Yunnan Province: Menglun Nature Reserve, secondary tropical seasonal moist forest (21.9164°N, 101.2830°E, 641–671 m a.s.l.), hand caught in leaf litter, 5–12.III.2007, leg. Guo Zheng. ***Paratypes***: 1♂ (IZCAS-Ar44635) and 2♀ (IZCAS-Ar44636, 44637), same data as holotype.

#### Etymology.

The specific name is dedicated to Mr Guo Zheng, the collector of this species; noun (name) in genitive case.

#### Diagnosis.

**Figure 7.**
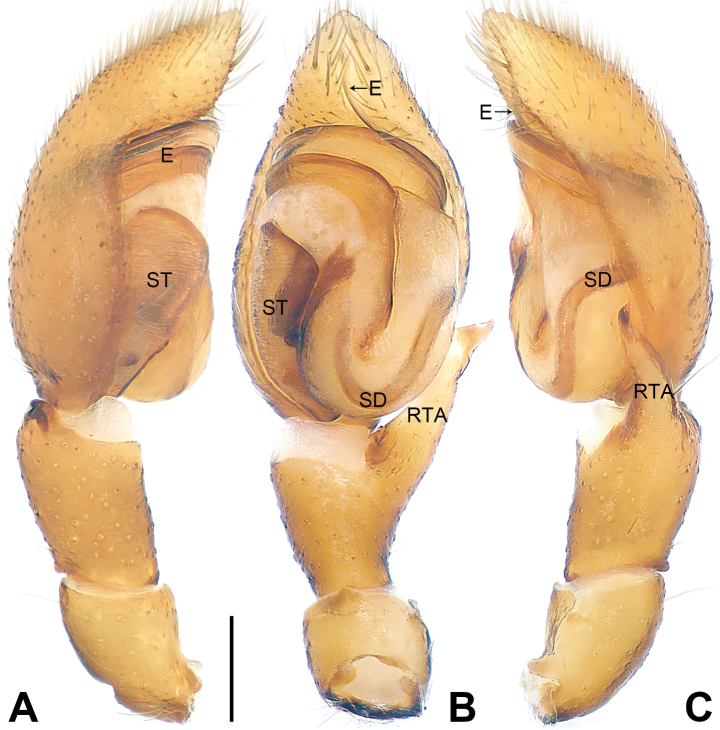
*Utivarachnazhengguoi* sp. nov., holotype male. **A–C** palp **A** prolateral view **B** ventral view **C** retrolateral view. Abbreviations: E = embolus, RTA = retrolateral tibial apophysis, SD = sperm duct, ST = subtegulum. Scale bar: 0.20 mm.

**Figure 8.**
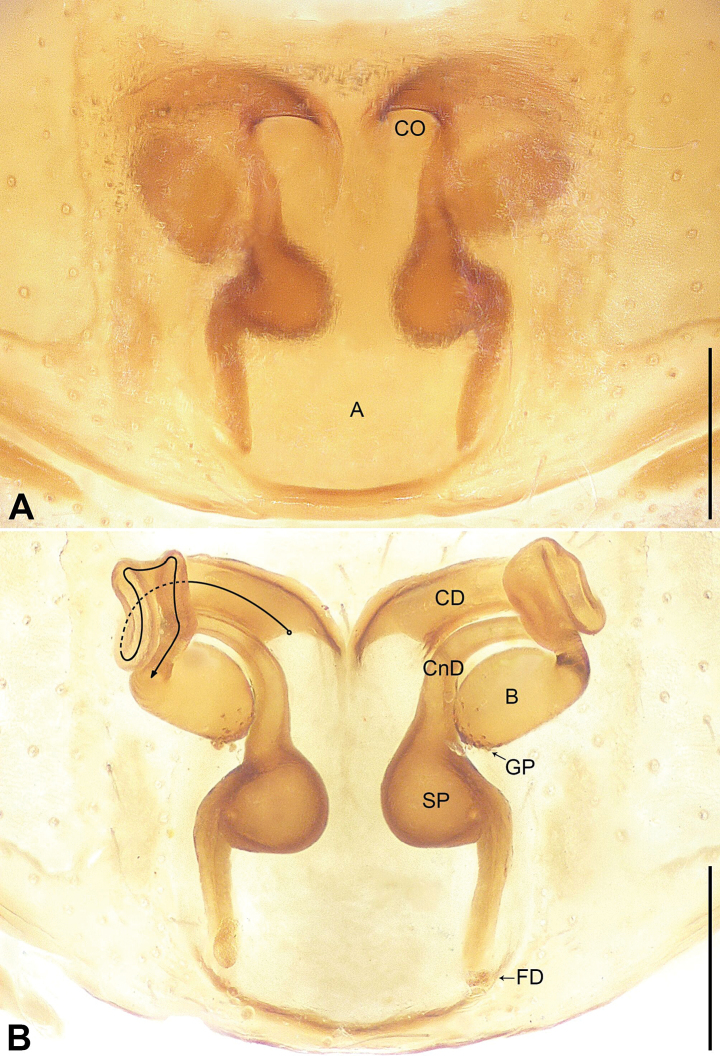
*Utivarachnazhengguoi* sp. nov., paratype female **A** epigyne, ventral view **B** vulva, dorsal view. Abbreviations: A = atrium, B = bursa, CD = copulatory duct, CnD = connecting duct, CO = copulatory opening, FD = fertilization duct, GP = glandular particles, SP = spermathecae. Scale bars: 0.20 mm.

**Figure 9.**
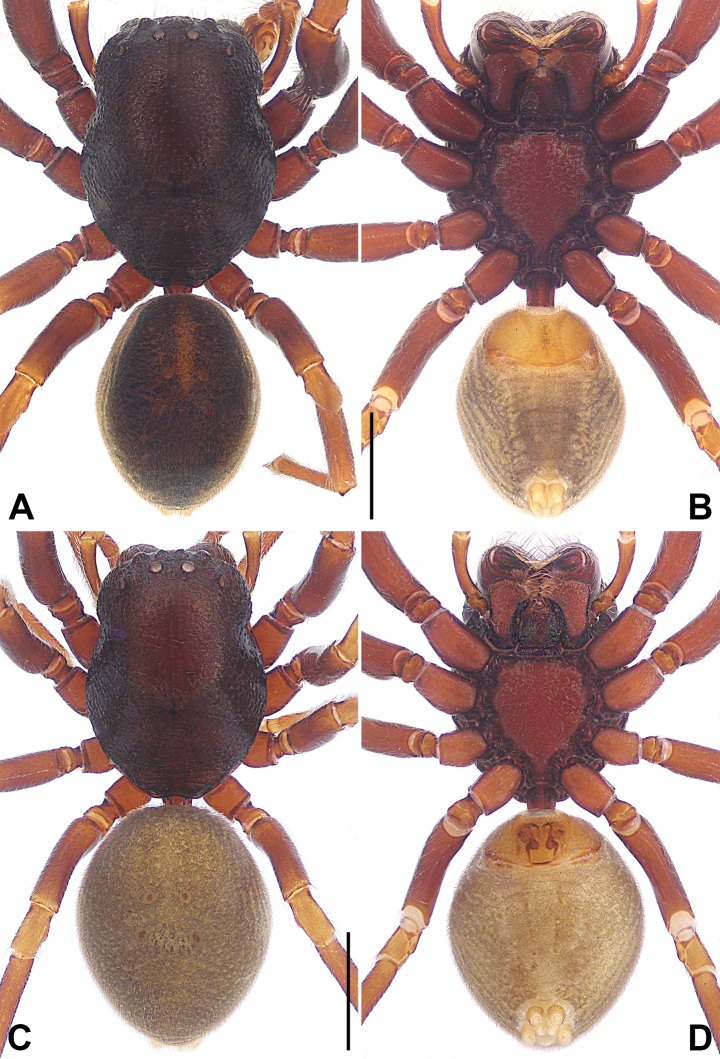
*Utivarachnazhengguoi* sp. nov., holotype male (**A, B**) and paratype female (**C, D**) **A–D** habitus **A** dorsal view **B** ventral view **C** dorsal view **D** ventral view. Scale bars: 1.00 mm.

Males resembles *U.fabaria*, *U.lata*, *U.rama* Chami-Kranon & Likhitrakarn, 2007, *U.subfabaria* Liu, Xu & Haddad, 2020, and *U.linyejiei* sp. nov. (cf. Figs 7, 9A, B and Jin et al. 2015: 573, figs 4A, C, D, 5A–D, 6C–E; Jin et al. 2015: 570, figs 1A, C, D, 2A–D, 3D–F; Chami-Kranon et al. 2007: 60, figs 1,4, 10–13, 14–17; Liu et al. 2020: 90, figs 1–3, 6A–D; Figs 1, 3A, B) by having similar long RTA, wide at the base, narrow at the end, and having a hook-shaped tip pointing anteriorly (Fig. 7B, C), but males of this species can be distinguished by the elliptical bulb, embolus (E) slightly shorter than widest part of bulb (Fig. 7B; vs bulb droplet-shaped, anterior part about half of posterior part, embolus about half of widest of bulb in *U.fabaria*; bulb almost square, embolus about as wide as widest of bulb in *U.lata*; bulb elliptical, embolus obviously shorter than widest of bulb in *U.rama*, *U.subfabaria* and *U.linyejiei* sp. nov.), by the subtegulum (ST) occupying approximately one-third of tegulum width, 3/5 of tegulum length in ventral view (Fig. 7B; vs 1/4 of tegulum width, 5/6 of tegulum length in *U.fabaria*; 1/4 of tegulum width, 4/5 of tegulum length in *U.rama*; 1/5 of tegulum width, 5/6 of tegulum length in *U.subfabaria*; 1/5 of tegulum width, 4/7 of tegulum length in *U.linyejiei* sp. nov.), and by the sperm duct (SD) extending to the base of tegulum (Fig. 7B; vs sperm duct separated from the base of tegulum by nearly three times the width of the sperm duct in *U.fabaria* and *U.rama*). Females resemble *U.arcuata* Zhao & Peng, 2014 (cf. Figs 8, 9C, D and Zhao and Peng 2014: 579, figs 3, 4) in having similarly large copulatory openings (CO) (Fig. 8A) and an atrium (A) occupying about 5/6 of the length of the epigyne (Fig. 8A), but they can be distinguished by their approximately straight copulatory openings (Fig. 8A; vs copulatory openings strongly curved, arch-shaped in *U.arcuata*), by the copulatory ducts (CD) with two or three sharp twists (Fig. 8B; vs one or two twists in *U.arcuata*), by the bean-shaped bursae (B) (Fig. 8B; vs S-shaped bursae, anterior part strongly restricted and curved in *U.arcuata*), and by the spermathecae (SP), which are separated by about half of their diameter (Fig. 8B; vs spermathecae separated by less than half of their diameter in *U.arcuata*).

#### Description.

**Male.** Habitus (Fig. 9A, B). Total length 4.68. Carapace (Fig. 9A): length 2.54, width 1.84, dark reddish brown; cervical groove, radial grooves and fovea indistinct; fovea short and dark. Eyes (Fig. 9A): AER procurved, PER recurved in dorsal view, PER wider than AER. Eye sizes and interdistances: AME 0.10, ALE 0.11, PME 0.10, PLE 0.11; AME–AME 0.10, AME–ALE 0.15, PME–PME 0.21, PME–PLE 0.31; MOA 0.31 long, anterior width 0.28, posterior width 0.42. Mouthparts (Fig. 9B): chelicerae dark reddish brown, with three promarginal (middle largest) and four retromarginal (proximal largest, distal smallest) teeth; endites depressed posteriorly, slightly convergent anteriorly, with dense setae on inner margin; labium nearly trapezoidal, length 0.50, width 0.41. Sternum (Fig. 9B) length 1.27, width 0.98, reddish brown with dark, reddish-brown edges, with precoxal triangles and intercoxal extensions, posterior region protruding strongly between coxae IV. Pedicel cylindrical, sclerotized, relatively short, reddish brown. Abdomen (Fig. 9A, B) yellow, 2.14 long, 1.57 wide, dorsum with scutum covering entire dorsal surface, with four central, indistinct, reddish-brown spots; venter with brown stripes. Spinnerets surrounded by brown rings. Legs: deep reddish brown, anterior legs distinctly thicker than posterior legs. Leg measurements: I 5.36 (1.52, 0.76, 1.24, 1.09, 0.75); II 4.99 (1.45, 0.69, 1.10, 1.08, 0.67); III 3.86 (1.15, 0.59, 0.67, 1.00, 0.45); IV 5.27 (1.43, 0.66, 1.14, 1.45, 0.59).

***Palp*** (Fig. 7A–C): tibia shorter than half of cymbium length; RTA about 1.06 times longer than tibia, with wide base and narrow, blunt tip, straight section subdistally to distally, slightly curvature distally. Bulb elliptical, wider in middle part; tegulum approximately 1.34 times as long as its maximum width in ventral view; subtegulum (ST) sclerotized, occupying approximately 1/3 of tegulum width in ventral view; sperm duct (SD) distinct, U-shaped in ventral view, extending to the base of tegulum. Embolus (E) long, anticlockwise, obliquely coiled twice, coils as wide as tegulum; basal portion of embolus lamellar, wide, arising at 12:00–12:30 o’clock from bulb; terminal portion of embolus filiform, resting in distal cymbial alveolus.

**Female.** Habitus (Fig. 9C, D). As in male except as noted. Total length 4.18. Carapace length 2.03, width 1.54, deep reddish brown, with lighter area at middle. Eye (Fig. 9C) sizes and interdistances: AME 0.09, ALE 0.11, PME 0.10, PLE 0.11; AME–AME 0.07, AME–ALE 0.10, PME–PME 0.17, PME–PLE 0.23; MOA 0.26 long, anterior width 0.25, posterior width 0.37. Mouthparts (Fig. 9D): chelicerae with three promarginal (middle largest) and four retromarginal (proximal largest, distal smallest) teeth. Sternum (Fig. 9D) length 1.06, width 0.85. Abdomen (Fig. 9C, D): length 2.15, width 1.63, dorsum with brown stripes and four distinct, reddish-brown spots centrally; venter with two lines of spots. Leg measurements: I 4.43 (1.25, 0.63, 1.02, 0.90, 0.63); II 4.07 (1.18, 0.60, 0.86, 0.88, 0.55); III 3.26 (0.96, 0.48, 0.63, 0.81, 0.38); IV 4.43 (1.18, 0.54, 1.00, 1.20, 0.51).

***Epigyne*** (Fig. 8A, B): epigynal plate longer than wide, spermathecae (SP) and bursae (B) distinct in ventral view. Atrium (A) large and nearly trapezoidal, occupying about 5/6 length of epigyne, posterior margin wider than anterior margin. Copulatory openings (CO) large, semicircular, located at anteriorly, separated by about their diameter. Copulatory ducts (CD) long, anterior part wide and posterior part narrow; copulatory ducts convoluted posteriorly, with two or three sharp twists. Connecting ducts (CnD) thinner than copulatory ducts, located on the lateral areas of copulatory openings, separated by more than spermathecae diameter. Bursae elliptical, separated by about 1.5 times their diameter; bursae with several small clusters of glandular particles (GP) on posterior surface, occupying about 1/5 of bursa diameter. Spermathecae globular, separated by less than their diameter. Fertilization ducts (FD) laminar, separated from each other by posterior width of atrium.

#### Distribution.

**Figure 10.**
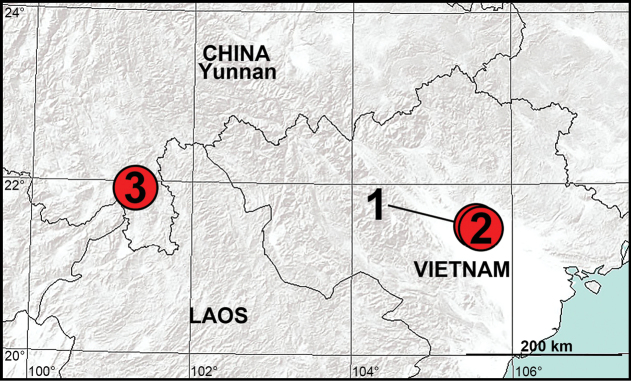
Distribution records of new *Utivarachna* species from China and Vietnam 1 = *U.linyejiei* sp. nov., 2 = *U.tamdao* sp. nov., 3 = *U.zhengguoi* sp. nov.

China (Yunnan, type locality; Fig. 10).

## Supplementary Material

XML Treatment for Utivarachna

XML Treatment for Utivarachna linyejiei

XML Treatment for Utivarachna tamdao

XML Treatment for Utivarachna zhengguoi

## ﻿Additional information

### Author ORCIDs

Chang Chu https://orcid.org/0000-0003-3520-5463

Shuqiang Li https://orcid.org/0000-0002-3290-5416

Dinh-Sac Pham https://orcid.org/0000-0001-8594-5270

Zhiyuan Yao https://orcid.org/0000-0002-1631-0949

### Data availability

All of the data that support the findings of this study are available in the main text.
